# Reproductive parameters of lambs immunocastrated with anti-GnRH vaccine

**DOI:** 10.1590/1984-3143-AR2020-0237

**Published:** 2021-06-21

**Authors:** Laiara Fernandes Rocha, Rosileia Silva Souza, Ana Lúcia Almeida Santana, Diego Silva Macedo, Ariadne Marques Silva Santana, Roberta Carvalho da Silva, Poliana Almeida Bezerra, Ronival Dias Lima de Jesus, Larissa Pires Barbosa

**Affiliations:** 1 Centro de Ciências Agrárias, Ambientais e Biológicas, Universidade Federal do Recôncavo da Bahia, Cruz das Almas, BA, Brasil

**Keywords:** anti-GnRH vaccine, azoospermia, testicular biometry, testosterone

## Abstract

The objective of this study was to evaluate the testicular biometric, seminal, and plasma testosterone levels in lambs subjected to an anti-GnRH vaccine as a method of castration. Thirty entire, crossbred Santa Inês male lambs were randomly distributed into three treatment (T): T1 was the control group, with the administration of 1 mL of saline solution subcutaneously (SC); 1.0 and 0.5 mL of an anti-GnRH vaccine were administered SC in T2 and T3, respectively. Testicular biometric variables, physical and morphological variables of semen, and plasma testosterone concentrations were evaluated. At D60, there was a reduction in testicular length, width, thickness, and scrotal circumference of the immunocastrated animals regardless of the vaccine dose used (*P* < 0.05). A reduction in semen physical variables at both dosages (*P* < 0.05) was observed, with azoospermia, in 80% and 70% of animals in the T2 and T3 groups, respectively. At D60, the immunocastrated animals also showed an increase in spermatozoa defects (*P* < 0.05), whereas plasma testosterone concentration decreased (*P* < 0.05). Immunocastration of lambs using the Bopriva vaccine at doses of 1.0 and 0.5 mL is efficient in inducing azoospermia in up to 80% of animals, although two doses in a 30-day interval are necessary for it to be an effective and safe method. Efficacy was demonstrated through a reduction in serum testosterone levels, testicular biometry, and seminal fluid analysis. Considering the efficacy of both doses in this study, we recommend using the lower dose (0.5 mL), which will allow for a 50% reduction in vaccine costs.

## Introduction

Immunocastration occurs when the animal’s immune system is stimulated to produce specific antibodies against the endogenous gonadotropin-releasing hormone (GnRH) (Tesema et al., 2019). Through this immunization, endogenous GnRH is neutralized, causing indirect suppression of luteinizing hormone (LH), follicle-stimulating hormone (FSH), and gonadal steroid release, thus, affecting spermatogenesis (Han et al., 2015).

This technique has been shown to be an effective alternative to manual castration in animal production systems (Thompson, 2000), where males are routinely castrated for social and sexual behavior control and to improve carcass quality. Castration by the complete removal of testes causes temporary pain and stress to the animals and may temporarily decrease animal performance (Melches et al., 2007). In addition, complete testes removal contravenes two of the five freedoms that govern animal welfare (freedom from pain, injury, or disease and fear and distress).

Thus, immunocastration is an alternative to conventional surgical sterilization because it is practical and noninvasive. This is achieved by administering a vaccine that promotes the blockage of GnRH release by the hypothalamus, interrupting normal testicular function and resulting in the suppression of spermatogenesis and reduced serum testosterone levels (Needham et al., 2017).

Immunocastration has been proven to be effective, and several specific vaccines are available on the market for swine (Dunshea et al., 2001; Zeng et al., 2002) and bovines (Amatayakul-Chantler et al., 2013; Pérez-Linares et al., 2017). Although anti-GnRH vaccines have already been studied in ovine (Han et al., 2015; Aponte et al., 2018) and caprine species (Lents et al., 2018), there is still no specific vaccine for these species on the market, and a vaccination protocol is lacking.

Different results have been obtained for different administration protocols and vaccination strategies for immunocastration in sheep (Needham et al., 2016; Aponte et al., 2018). Therefore, it is essential to establish an effective vaccination protocol capable of promoting an adequate response while maintaining animal performance and improving carcass characteristics and meat quality (Needham et al., 2017). Therefore, the objective of this study was to evaluate the testicular biometric, seminal, and plasma testosterone levels in lambs subjected to an anti-GnRH vaccine as a method of castration.

## Methods

The study was conducted on the Corcovado Farm, located in the Jaguara district in the municipality of Feira de Santana/Bahia, situated at 12°07′22.4” S latitude and 39°06′33.5” W longitude. According to the Köppen classification (type Aw), the area has a tropical climate, with an average temperature and relative humidity of 24.2 °C and 81%, respectively, and an annual rainfall of 820 mm (Station A413 INMET).

Thirty entire crossbred Santa Inês male lambs aged 4.19 ± 0.41 months were used, with an average body condition score of 3.00 ± 0.37 (Russel et al., 2016). The animals were randomly allocated to three treatments (T) in a completely randomized design. The control treatment group, T1 (*n* = 10), was administered 1.0 mL of physiological saline solution subcutaneously (SC). For the vaccine-administered groups, 1.0 and 0.5 mL of the anti-GnRH vaccine was administered SC in T2 (*n* = 10) and T3 (*n* = 10), respectively. Animals in T2 and T3 received a second dose of the vaccine 30 days after the first injection, while the animals in T1 received a second physiological saline solution injection at the same time.

The animals were subjected to an extensive management system during the experimental period, which was 60 days, grazing on *Urochloa mosambicensis* pasture, with *ad libitum* access to water and mineral blocks (Ovinofós, Tortuga, Brazil). The animals were evaluated regularly for their health and reproductive status.

For immunization, the Bopriva commercial vaccine (Pfizer Animal Health, São Paulo, Brazil) was used, where each milliliter of vaccine provided 400 µg of GnRH conjugate and a carrier protein. Evaluation of testicular biometry and the physical and morphological aspects of semen were evaluated at second injection (D30), and 30 days after the second injection (D60), and plasma testosterone concentration at D60.

The following were measured to evaluate testicular biometry: length (measured in the dorsoventral direction of each testis, excluding the tail of the epididymis), thickness (the longest craniocaudal distance of each testicle), right and left testicular widths, and scrotal circumference (measured at the middle of the testicles, at the point of the greatest horizontal dimension, involving the two testicles and the scrotal skin). The right and left testicular widths were measured using a pachymeter (Digimess, São Paulo, Brazil), and the scrotal circumference was measured using a measuring tape.

Semen production and quality were evaluated by collecting semen using an electro-ejaculator (Autojac M, Neovet, Uberaba, Brazil). Immediately after collection, the ejaculate was subjected to physical evaluation (seminal volume, mass motility, progressive sperm motility, sperm vigor, and sperm concentration) and morphological evaluations (major, minor, and total defects), according to the Brazilian College of Animal Breeding (CBRA, 2013).

Blood samples were collected to determine the plasma concentration of testosterone by venipuncture of the jugular vein and collection in vacutainer tubes with EDTA as an anticoagulant. Vacutainers were refrigerated until centrifugation (1200 × *g* for 12 min). The plasma was kept frozen (-20 °C) until the hormonal quantification was performed by chemiluminescence using a commercial kit (Access Testosterona; Beckman Coulter, Pasadena, CA, USA) following the guidelines recommended by the manufacturer.

The exchange rate between the Brazilian real (R$) and US dollars (US$) was calculated to determine the cost of the protocols according to the commercial dollar quotation disclosed by the Banco Central do Brasil at the time and the retail value of hormones in Salvador Bahia (Table 1).

**Table 1:** Dollars cost (US$) of the vaccine used to immunocastrate lambs, in Salvador Bahia city, in January of 2019.

**Vaccine**	**Bopriva**	**Total cost of each protocol**
Price per bottle ($) 50 mL	162.73	
Price per dose ($) 1.0 mL	3.25	T2 6.51 (using 2 doses of 1.0 mL each)
Price per dose ($) 0.5 mL	1.62	T3 3.25 (using 2 doses of 0.5 mL each)

Quotation and conversion from real (R$) to US dollar (US$) performed in the website of Banco Central do Brasil. Value of a dollar in the month January of 2019= 3.6869 reais.

The data were subjected to a normality evaluation using the Shapiro–Wilk test. For variables that presented a normal distribution, ANOVA and Tukey’s test were performed. For nonparametric variables, the Kruskal-Wallis test was performed at a 5% level of significance. The Statistical Package for the Social Sciences (SPSS) program (version 23, 2015) was used for these analyses.

The study was approved by the Ethics Committee on the Use of Animals (CEUA) of the Universidade Federal do Recôncavo da Bahia, under protocol number 23007.010944/2018-68.

## Results

### Testicular biometry

There was no difference among the treatment groups for testicular biometric variables (*P* > 0.05) at D30. However, at D60 after anti-GnRH vaccination, all testicular biometric variables presented a difference among treatments (*P* < 0.05) (Table 2), with the immunocastrated groups (1.0 and 0.5 mL) showing a reduction in the measured variables. Notably, the effect of vaccination was apparent after the second dose of the anti-GnRH vaccine in both treatments.

**Table 2:** Testicular biometry of lambs immunocastrated with the anti-GnRH vaccine.

**Variables (cm)**	**Treatments**				***P* Value**
	**Control**	**1.0mL of the Vaccine**		**0.5mL of the Vaccine**	
Thirty days after the first application of the anti-GnRH vaccine (D30)					
Scrotal Circumference1	22.10 ± 5.93	20.84 ± 5.31	20.79 ± 4.71		0.860
Testicular Length^1^	5.57 ± 1.92	4.97 ± 1.56	4.95 ± 1.40		0.703
Testicular Width^1^	3.98 ± 1.25	3.69 ± 1.10	3.82 ± 1.11		0.842
Testicular Thickness^1^	3.97 ± 1.39	3.64 ± 1.10	3.77 ± 1.09		0.820
Thirty days after second dose of the anti-GnRH vaccine (D60)					
Scrotal Circumference2	25.50 ± 7.75^a^	17.50 ± 5.65^b^	18.50 ± 6.60^b^		0.040
Testicular Length^1^	6.06 ± 2.00^a^	3.84 ± 0.79^b^	4.02 ± 1.00^b^		0.003
Testicular Width^1^	4.05 ± 1.12^a^	2.94 ± 0.69^b^	3.05 ± 0.74 ^b^		0.023
Testicular Thickness^1^	4.17 ± 1.24^a^	2.98 ± 0.82^b^	3.11 ± 0.78^b^		0.027

^1^Data refer to the mean ± standard deviation of the mean, and distinct letters on the line, indicate statistical difference by the Tukey test. ^2^Data refer to the median ± interquartile range, and different letters on the line, indicate statistical difference by the adjusted Kruskal-Wallis test. For both tests, the 5% significance level was adopted.

### Production and seminal fluid quality

There was no significant difference among the treatment groups in physical and morphological semen evaluations (*P* > 0.05) at D30 (Table 3). Corroborating the testicular biometry results, a second dose of the anti-GnRH vaccine was needed to effectively promote physiological effects of immunocastration. At D60 after anti-GnRH vaccination, there was a significant difference (*P* < 0.05) in the seminal volume, mass motility, progressive sperm motility, sperm vigor, sperm concentration/mL, total sperm concentration, and the total major and minor defects between the control and the immunocastration treatments (Table 3).

**Table 3:** Physical and morphological semen evaluation of lambs immunocastrated with the anti-GnRH vaccine.

**Variables**	**Treatments**			***P* Value**
	**Control**	**1.0mL of the Vaccine**	**0.5mL of the Vaccine**	
Thirty days after the first application of the anti-GnRH vaccine (D30)				
Seminal volume (mL)^2^	0.200 ± 0.50	0.300 ± 0.50	0.200 ± 0.55	0.543
Mass motility (0-5)^1^	2.40 ± 2.27	1.00 ± 1.22	2.00 ± 1.58	0.571
Motility (%)^2^	85.00 ± 95.00	70.00 ± 62.50	80.00 ± 52.50	0.958
Vigor (0-5)^1^	2.60 ± 2.50	2.00 ± 1.54	2.90 ± 1.63	0.852
Concentration (x10^9^/mL)^1^	1.22 ± 1.82	0.87 ± 1.33	0.43 ± 0.56	0.399
Total Concentration (x10^6^)1	274.45±400.57	265.72±404.71	196.75±426.69	0.906
Minor Defects (%)^2^	10.00 ± 40.00	51.00 ± 37.25	56.50 ± 25.75	0.336
Major Defects (%)^1^	28.00 ± 28.84	32.00 ± 24.27	18.00 ± 9.61	0.599
Total Defects (%)^1^	49.40 ± 30.69	79.50 ± 13.59	66.00 ± 27.57	0.182
Thrity days after the second dose of the anti-GnRH vaccine (D60)				
Seminal Volume (mL)2	0.225 ± 0.25^b^	0.500 ± 0.63^a^	0.300 ± 0.35^b^	0.049
Mass motility (0-5)^2^	0.50 ± 3.00^a^	0.00 ± 0.00^b^	0.00 ± 0.00^b^	0.003
Motility (%)^2^	85.00 ± 90.00^a^	15.00 ± 0.00^b^	35.00 ± 0.00^b^	0.020
Vigor (0-5)^2^	0.75 ± 3.90^a^	0.00 ± 0.00^b^	0.00 ± 1.10^b^	0.034
Concentration (x10^9^/mL)^2^	222.5 ± 816.2^a^	0.00 ± 12.50^b^	0.00 ± 136.5^b^	0.020
Total Concentration (x10^6^)^2^	63.12 ± 215.75^a^	0.00 ± 2.50^b^	0.00 ± 43.09^b^	0.036
Minor Defects (%)^2^	14.50 ± 9.00	52.25 ± 0.00	51.00 ± 0.00	0.145
Major Defects (%)^1^	9.28 ± 5.74^a^	44.00 ± 26.87^b^	36.00 ± 6.25^b^	0.003
Total Defects (%)^2^	24.00 ± 15.50^a^	96.25 ± 0.00^b^	83.00 ± 0.00^b^	0.035

^1^Data refer to the mean ± standard deviation of the mean, and distinct letters on the line, indicate statistical difference by the Tukey test. ^2^Data refer to the median ± interquartile range, and different letters on the line, indicate statistical difference by the adjusted Kruskal-Wallis test. For both tests, the 5% significance level was adopted.

### Plasma concentration of testosterone

**Figure 1:**
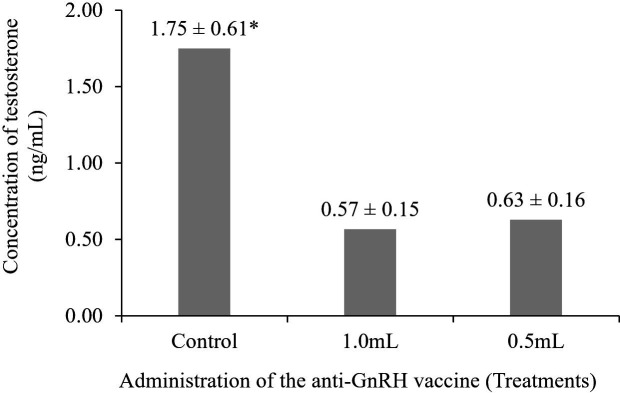
Plasma concentration of testosterone. The data refer to the mean ± standard deviation of the mean, and the mean followed by the asterisk (*) indicates a difference between the control group and the immunocastrated groups (1.0 or 0.5 mL) by the Tukey’s test, at a 5% level of significance.

There was a significant difference among the treatment groups (*P* < 0.05) in plasma testosterone concentration at D60 (Figure 1), with a reduction in concentration in the immunocastrated animals. At the same time, the plasma testosterone concentration in the control group remained within the normal range.

## Discussion

### Testicular biometry

At D30, variables were within the recognized values for the species and age, with the same scrotal circumference (20.84 ± 3.22 cm), according to CBRA (2013). Similarly, the length (5.83 cm), width (3.11 cm) (Alves et al., 2006), and testicular thickness (3.9 ± 0.1 cm) (Saaed and Zaid, 2019) were previously reported, demonstrating that the first injection, regardless of dose, did not affect the testicular biometry of lambs. According to the manufacturer’s recommendations (Bopriva), a second vaccine dose is necessary to promote bovine immunocastration. The first injection only sensitizes the animal’s immune system, and it produces an effective immune response by increasing antibody production at 7–14 days after the second injection.

At D60, treatment differences in testicular biometrics were apparent. The scrotal circumference of 25.50 ± 7.75 cm in the control group was within the average values for the species and age. The scrotal circumference in the control animals was similar to the average scrotal circumference of 26.29 ± 2.14 cm reported by Pinto et al. (2016) and the results of Gokdal et al. (2010), who demonstrated that the scrotal circumference of immunized sheep differed from that of the control group and remained smaller during the study (*P* < 0.01).

The reduction in scrotal circumference in the treated groups resulted from a decrease in testicular width and thickness by approximately 21% and 18%, respectively. Testicular length decreased by 29% until 60 days after the first anti-GnRH vaccination, regardless of dosage.

The testicular length of 6.06 ± 2.00 cm in animals from the control group was within the average values for the species and age and similar to that reported by Han et al. (2015b; 6.42 ± 0.74 cm). The reduction in testicular length of immunocastrated lambs was similar to that reported by Wassie et al. (2019), who used a kisspeptin-54-based vaccine to perform immunocastration in sheep and demonstrated a significant (*P* < 0.05) reduction in testicular length in the immunized group.

While testicular width in the control group increased during the study, testicular width in lambs from the immunized groups decreased after the second vaccine dose. Han et al. (2015b) evaluated the effect of vaccines in lamb immunocastration and found testicular widths of 4.25 ± 0.53 cm in the control group and 3.55 ± 0.24 cm in the immunized group. Similarly, Wassie et al. (2019) obtained a testicular width reduction from 5.33 ± 0.10 cm in the control group to 4.75 ± 0.05 cm in the immunized group. The strategy of immunocastration in the current study greatly reduced testicular width, and the use of both 1.0 mL and 0.5 mL of Bopriva was effective in reducing testicular width.

The average testicular thickness in the immunized groups also decreased after the second vaccine dose. In contrast, the control group presented a testicular thickness of 4.17 ± 1.24 cm within the value expected for the species and similar to that reported by Saaed and Zaid (2019; 4.2 ± 0.2 cm) in lambs of the same age.

### Seminal fluid production and quality

There was no difference in the seminal fluid parameters between the control and treatment groups at D30. This observation is consistent with the vaccine only sensitizing the immune system without sufficient antibodies. According to Amatayakul-Chantler et al. (2013), immunocastration generally occurs 14 days after the vaccine booster, concomitant with an increase in antibody production. The seminal fluid responses in the current study were similar to those in Santa Inês sheep at 5 months of age.

Fraga et al. (2015) observed that sperm production commenced at an average age of 4.71 ± 0.36 months in sheep, presenting a mass motility of 1.0 ± 1.73; motility of 65.00% ± 39.05%, and sperm concentration of 0.75 ± 0.12 × 10^9^ sperm/mL. In the current study, lambs at 5.19 months of age presented average values of 1.8 ± 1.69, 78.33% ± 70%, and 0.84 ± 1.23 × 10^9^ sperm/mL in mass motility, motility, and sperm concentration, respectively, in their ejaculate. The pre-pubertal status can explain these differences. The seminal fluid composition is expected to be more predictable at sexual maturity.

Major, minor, and total defects were more than those expected in adult sheep, according to the CBRA (2013). Total defects cannot exceed 20%, but an average of 64.96% ± 23.95% was found in the present study. This value is higher than that reported by Pacheco et al. (2009) in pre-pubertal animals (47.2% ± 13.5% total defective sperm). In pre-pubertal animals, these numbers are expected to exceed the normal range. Upon reaching sexual maturity, the number of defects should decrease and be stabilized, as suggested by Pacheco et al. (2009).

The results obtained at D30 for testicular biometry and physical and morphological semen evaluation suggest that a second vaccine dose is necessary to promote immunocastration effectively. Lents et al. (2018) also used Bopriva (indicated for cattle) in goats and demonstrated that two vaccine doses were essential for effective immunocastration in goats. Amatayakul-Chantler et al. (2013) also suggested two vaccinations when using Bopriva in bovine. Schaut et al. (2018) agreed that immunocastrating vaccines require a booster dose to achieve a sufficient antibody titer to effect immunocastration.

The vaccine could effect immunization by decreasing the lambs’ reproductive function, possibly by producing specific endogenous anti-GnRH antibodies. The unavailability of endogenous GnRH alters the functioning of the hypothalamic–pituitary–gonadal axis and reduces testicular biometry and sperm production in sheep (Tesema et al., 2019).

The percentage of minor defects was not significantly different (*P* > 0.05) between the control and immunized sheep. However, the immunized groups showed reductions of 100%, 66.66%, 100%, 100%, and 100% in the mass motility, motility, sperm stamina, sperm concentration/mL, and total sperm concentration, respectively, between D30 and D60, with values far below those recommended by CBRA (2013) for sheep of this age.

The lambs that did not receive the vaccine showed a 51.41% reduction in the occurrence of abnormal sperm. Souza et al. (2010) found that defective spermatozoa decrease with age, and the primary defects occur with greater frequency at the pre-pubertal and pubertal stages. Lambs vaccinated in the current study, regardless of dose, showed a 23.28% increase in the number of abnormal spermatozoa. Frazão et al. (2014) obtained 24.30% ± 2.9% total sperm pathologies, similar to those of the control group in the current study. CBRA (2013) recommends that this value be lower than 20%.

According to Lima et al. (2013), this change in sperm morphology should decrease with life stage owing to the maturation of the seminiferous epithelium and consequent progressive stabilization of seminal characteristics, thus, reducing the percentage of defective spermatozoa.

At D60, there was a reduction in physical semen parameters in vaccinated animals (*P* < 0.05), with 80% and 70% azoospermia in the groups that received 1.0 and 0.5 mL, respectively. Lents et al. (2018) found azoospermia of 83.33% in goats immunocastrated with 1.0 and 0.5 mL of Bopriva.

Aponte et al. (2018) evaluated the effects of active anti-GnRH immunization on the reproductive function of lambs and observed that spermatozoa were not found in 60% of the transverse sections of seminiferous tubules in the immunized group. Ferro et al. (2004) also used an anti-GnRH vaccine in adult sheep, which had active spermatozoa in only 19% to 28% of the tubules in immunized animals, compared with the untreated group. Immunosuppression is, therefore, useful, although not through the mechanism of suppression of spermatogenesis (Ferro et al., 2004; Aponte et al., 2018; Lents et al., 2018).

Despite the lack of fertility tests in the present study, the results suggest that ejaculates are unable to achieve fertilization, primarily because of the observed azoospermia associated with low sperm motility and vigor, in addition to the increase in defects.

### Plasma testosterone concentration

In the present study, there was a 60% decrease in the plasma testosterone concentration in the immunized groups than in the control group. Amatayakul-Chantler et al. (2013) also observed a reduction of approximately 34% in testosterone levels with the same vaccine in bovines.

The plasma testosterone values at D60 when the animals were 6 months old were similar to those previously described for sheep. In the present study, a plasma testosterone value of 1.75 ng/mL was found in the control group, close to that reported by Nazari-Zenouz et al. (2016) in 6-month-old lambs (1.69 ng/mL).

Gokdal et al. (2010), Tesema et al. (2019), and Needham et al. (2019) investigated the effects of anti-GnRH immunocastration on the reproductive characteristics of lambs. Immunocastration reduced fertility, elevated anti-GnRH antibodies, suppressed testosterone production, and reduced testicular growth.

The reduction in testosterone supports the findings for testicular biometry and physical and morphological analysis of the sperm, with reduced values in immunocastrated animals. The low testosterone values suggest that the immunization effect through anti-GnRH antibody production causes endogenous GnRH neutralization and consequently limits the response of testicular receptors to gonadotropins (Janett et al., 2012).

## Conclusion

Immunocastration in lambs using the Bopriva vaccine at doses of 1.0 and 0.5 mL is efficient in inducing azoospermia in up to 80% of animals; however, two doses at an interval of 30 days are necessary for it to be an effective and safe method. Efficacy was demonstrated through the reduction of serum testosterone levels, testicular biometry, and seminal fluid analysis. A lower dose (0.5 mL) is recommended as it is equally effective and allows for a 50% reduction in vaccine costs.
